# Nursing Students’ Satisfaction: A Comparison between Medium- and High-Fidelity Simulation Training

**DOI:** 10.3390/ijerph18020804

**Published:** 2021-01-19

**Authors:** Ana Rosa Alconero-Camarero, Carmen María Sarabia-Cobo, María José Catalán-Piris, Silvia González-Gómez, José Rafael González-López

**Affiliations:** 1 Department of Nursing, University of Cantabria, Avenida Valdecilla, s/n, 39008 Santander, Spain; alconear@unican.es; 2 IDIVAL—Instituto de Investigación Sanitaria Valdecilla, s/n, Calle Cardenal Herrera Oria, 39011 Santander, Spain; 3 Department of Nursing, Faculty of Nursing, Physiotherapy and Podiatry, Universidad de Sevilla, C/Avenzoar nº6, 41009 Seville, Spain; jcatalan@us.es (M.J.C.-P.); joserafael@us.es (J.R.G.-L.); 4 Cantabrian Health Service, Av. Herrera Oria, s/n, 39011 Santander, Spain; silvia.gonzalezg@scsalud.es

**Keywords:** simulation training, nursing students, personal satisfaction, high-fidelity simulation training

## Abstract

Training based on clinical simulation is an effective method of teaching in nursing. Nevertheless, there is no clear evidence about if it is better to use high- or medium-fidelity simulation. The aim is to analyse if students are more satisfied when their clinical simulation practices are based on high-fidelity simulation (HFS) or medium-fidelity simulation (MFS). Students´ satisfaction was assessed using the Satisfaction Scale Questionnaire with High-Fidelity Clinical Simulation. The sample is composed of 393 students from two Spanish Universities. Satisfaction with simulation in nursing students is significantly greater in MFS than HFS. Simulation is beneficial for learning in all its forms, but for the acquisition of basic skills, and at a lower cost, MFS proves to be effective. However, high-fidelity is not always better than medium-fidelity as this depends on the student’s level of knowledge and clinical experience.

## 1. Introduction

The educational model of the European Higher Education Area recognises the fundamental role of professional competencies for student learning [1]. In Spain, Nursing Degrees are designed by a 240 European Credit Transfer and Accumulation System (ECTS) [2] (1 ECTS is equivalent to 25 h, an academic course has 60 ECTS): 180 ECTS is recommended as the minimum of theory credits and 90 ECTS for practice credits and the Degree Project to achieve a professional degree [2].

However, being competent in a specific area is complex because students must be able to integrate knowledge, skills, capabilities, attitudes and learning outcomes, all of which enable problem solving in various ‘real-life’ situations [3,4,5]. In this sense, simulation as an optimal teaching tool may help one achieve the competencies of preparing students for clinical practice, together with other teaching methods [6,7,8,9].

Clinical simulation complements clinical rotations in real clinical contexts—in Spain, this usually begins in the second year—rather than acting as a substitute for them, as real experiences have certain nuances which are not reproducible in a simulated environment [10]. Numerous studies have suggested that high-fidelity simulation (HFS) is a realistic teaching method which boosts knowledge [11,12,13]. The conceptual model by Benner (1984) [14] is used to determine the skills and knowledge needed in simulation, and for developing students’ competency and critical thinking. During the simulation activities, students of all disciplines can collaborate and interact in a safe environment in order to undergo the transition from novice to expert. The acquisition of skills based on experience is safer and faster when it is based on a sound theoretical foundation [15]. Furthermore, five degrees of competencies can be identified: beginner, advanced beginner, competent, efficient and expert (adapted from the Dreyfus Model) [14]. Expert nurses know the patients, and consequently, they are able to identify their problems without wasting time and act based on a thorough knowledge of the overall situation. An essential aspect for the expert nurse is to understand the person’s real needs, although this may involve a real change in practice [16].

Considering this, training based on clinical simulation consists of replacing reality with a simulated scenario, that is as realistic as possible, in which students and professionals can learn communication skills, psychomotor skills and team-working skills [17]. These scenarios and the methodologies applied vary according to the skills to be trained and are always based on ensuring safety for both students as well as patients [18,19].

According to the specific technological complexity, the types of simulators are divided into three categories depending on ‘fidelity’ (the degree of realism of the models and the intended experience) [20]: low, medium and high. The complexity of low-fidelity simulation (LFS) is essential and is used to acquire basic psychomotor skills in a simple procedure or physical examination which only simulates one part of the body, for example, basic cardiac auscultation. The complexity of medium-fidelity simulation (MFS) offers more realism than LFS: the use of a mannequin connected to a less complex pre-established software which enables the instructor to manage basic physiological variables with the aim of achieving the development of competency. The objective is for the participant to be able to resolve problems, perform a skill and practice decision-making during a clinical scenario. Lastly, the complexity of HFS integrates multiple physiological variables for the creation of realistic clinical scenarios with life-size mannequins. Thus, HFS includes complements which provide the simulators with personality and enable the users to identify themselves with the same realism as real life. The aim is to train competencies and advanced techniques in the management of critical situations [17]. It is also important to bear in mind that the potential use of simulation in competency tests cannot be achieved until the educators and researchers acquire the necessary knowledge or skills to use these educational strategies, develop realistic case scenarios and design and validate methods of standardised and reliable tests [21].

Although students do not always recognise what is best for them [22], another relevant aspect of the simulation experiences highlighted in the literature is the level of satisfaction. In this sense, the learning process is a broader concept [23], and we only focus on the satisfaction level in the simulation. This teaching technique facilitates participation in learning and encourages performance and self-confidence [24]. In addition, it helps with both the development of skills and the acquisition of knowledge. HFS has great advantages [25,26,27], although the cost of these kinds of simulators is high [28]. Furthermore, technological complexity is an inherent obstacle to HFS [29]. In other words, HFS has a high cost, which not all universities can afford. Its orientation is indicated by the literature, especially for professionals or for postgraduate training. Although MFS does not allow the training of advanced skills, it does allow undergraduate students to acquire basic skills effectively. The search for cost-effective measures is also part of good teaching practice, so it is interesting to explore the effectiveness of MFS.

There are previous studies that tried to compare the different types of simulation with respect to the satisfaction that students obtained with their learning, but they are not conclusive [30,31]. There is a lack of consensus of scientific research on the satisfaction of nursing students comparing MFS and HFS [26,32,33]. For the above reasons, our research hypothesis states: Do differences exist in the level of satisfaction of nursing students regarding the use of HFS compared to MFS?

Considering these points, the aim of this manuscript is to analyse if students are more satisfied when their clinical simulation practices are based on HFS than on MFS. Our research aims to contribute to this debate due to the different simulator costs (high-fidelity vs. medium-fidelity). If there are no significant differences in the learning process, this could mean relevant cost savings. This is why this research has made some methodological improvements in the sample (larger, homogeneous—second year of studies—and including evidence from two Universities), considering some weaknesses of the previous literature.

## 2. Materials and Methods

### 2.1. Design and Participants

The study was designed and carried out as a quasi-experimental investigation. Students from the second academic year who enrolled in the simulation subjects (“Urgencies and emergencies in a special situation” in the University of Cantabria, and “Vital support” in the University of Seville) in the Nursing degree at Nursing Schools from two Universities in Spain (one from the north—the University of Cantabria—and another one from the south—the University of Seville) were invited to participate in the study.

A total population study (*n* = 401, 210 students from the University of Cantabria and 191 students from the University of Seville, which meant 100% participation) was proposed since it is well defined, accessible and not too large for data collection. This data collection was performed in 2017. After reading the information related to the study, they voluntarily confirmed their availability and interest in participating, being informed that their participation would have no effect whatsoever on their grades. All students were approached, although some of them refused to participate in the simulation activity or to complete the questionnaire (3 of the University of Cantabria and 5 of the University of Seville). The final sample was composed of 393 students (207 students from the University of Cantabria and 186 students from the University of Seville). Students from the University of Cantabria participated in an HFS intervention, while those enrolled in the University of Seville took part in an MFS intervention. The curriculum in both schools follows the guidelines of national requirements.

### 2.2. Interventions Design of Activities

The study followed the guidelines of the Consolidated Standards of Reporting Trials (CONSORT) for the reporting of this study [34].

At the University of Cantabria (HFS), theoretical classes were taught at the beginning of the course, accompanied by theoretical support in order to review the most important points designed for the assessment and intervention of patients in a critical status. Four different scenarios—previously peer reviewed and validated—were used in this study. Each scenario explained the situation of the patient, whether in the ward, the emergency room, at their home, or in a geriatric hospital. In each scenario, there were 4 or 5 students who performed the simulation with a professional role, while the remaining observers (fifteen) followed the evolution in an adjacent room on a TV monitor. All the students intervened in at least one of the cases. This was the first time that they had done so with an HFS.

To elaborate the verification list, we have considered the competencies which have been selected as being the most representative in the context of the management of the critical patient (patient-centred assessment airway): (A) ventilation, (B) circulation and (C) recognition of the critical event, application of basic or advanced vital support measures, re-assessment of the patient, knowledge regarding the administration of drugs, communication with the patient, where appropriate, and communication with the medical team. Previously, the professors had prepared the scenario, simulators, materials and necessary devices, as well as the patient’s clinical history.

The training took place at a simulation scenario in two rooms that were prepared for this purpose. The necessary staff included one instructor/professor (if the professor is an instructor, one less person is needed), a technician acting as an assistant within the room and a software technician. Simulation has been used as a learning method over fifty hours (twenty-five hours per academic year).

We used two types of computer-controlled high-fidelity mannequins (METIman^®^- Sarasota, FL, USA- and SimMan 3G^®^- Stavanger, Norway). Initially, on the first day of training in each group, a talk lasting approximately five minutes explained the aims and organisation of the virtual hospital, including specifics regarding the lockers for changing clothes and changing into a uniform. Thereafter, a specific scenario took place, previously explaining to the students and observers what they would find in each room. This meant that, in the event of a critical situation, the student could visualise a patient with an illness in a realistic work environment. The team of instructors, both within and outside the simulation room, were in continuous communication via the use of microphones and headsets and provided ongoing moment-by-moment information. Each situation was both audio- and video-recorded to enable, were it necessary, an analysis between the case instructor and the students. Lastly, the debriefing took place. This was what any simulation should include: all the skills can be worked on, such as team-work, decision-making, task management and other psychomotor skills [35]. The approximate duration of each case was 12 min, while the reflection or debriefing phase lasted approximately 20 min.

At the faculty in the south, the University of Seville, MFS was performed due to the lack of high-fidelity simulators. Simulation was used as a method of learning and for the assessment of the quality of the practice on basic cardiorespiratory resuscitation (CPR) performed by the students. Theoretical classes were taught at the beginning of the academic year (six hours), accompanied by the corresponding theoretical support for the follow-up and review of the most important aspects designed for the assessment and intervention in a patient in a critical status with cardiorespiratory arrest. At the beginning of the course, and while the theoretical classes were being taught, MFS was performed. The scenarios and the verification lists were similar to those of the school in the north.

The cardiorespiratory resuscitation (CPR) manoeuvres were practised between April and May 2017. This was done by using visual feedback simulation (Resusci^®^Anne SKillreporte™ Laerdal^®^ mannequin) to perform the CPR manoeuvres on four practical cases using the simulation mannequins. This was performed in small groups of 10–15 students, taught in three teaching units at three different hospitals. Three teachers participated in the MFS practice. The ‘supposed’ patient was found in a supine position, on the floor, having suffered an apparent cardiorespiratory arrest. Each student had to perform a focused assessment of the person and perform CPR during three complete cycles (1 cycle: 30 compressions/2 ventilations), and in no case was the sequence corrected or interrupted until the resuscitator had finished their practice or the case. All the students simulated with the simulation mannequin with feedback, in order to quantify the quality of the external cardiac massage and the ventilations performed. This assessment was completed with the direct observation on behalf of the teacher, using a verification checklist proposed by the national CPR plan to assess the appropriate adherence to the recommendations given in the resuscitation guidelines.

The duration of the training in each group was 30 min with the Resusci^®^Anne SKillreporte™ Laerdal^®^ simulator with feedback in order to correct errors detected in the first report via direct visualisation of the effectiveness of the compressions and ventilation performed on the self-assessment mannequin. The debriefing consisted of the overview of each student’s report to identify the errors made, relating these with their possible causes and identifying the possible solutions.

### 2.3. Instruments Used for Data Collection

For measuring the satisfaction of the students with the training, the “Satisfaction Scale Questionnaire with High-Fidelity Clinical Simulation—SSHF” [36] was used. The questionnaire is composed of 38 closed questions valued by a 5-degree Likert scale, and structured in eight domains: (1) simulation utility, (2) characteristics of cases and applications, (3) communication, (4) self-reflection on performance, (5) increased self-confidence, (6) relation between theory and practice, (7) facilities and equipment and (8) negative aspects of simulation. The greater the score for each scale, the greater the level of agreement with it.

This questionnaire evaluates student satisfaction with HFS; however, it is known that HFS is costly, and that MFS helps lower the economic burden and may be equally effective. This is why we decided to assess both types of fidelity with the same questionnaire.

### 2.4. Ethical Considerations

The approval of the Ethics Committee of Projects at the University of Cantabria was obtained (CE 06/2017) as well as the authorisation of the other participating centre (the University of Seville). The students were informed of the study and granted their consent. Confidentiality was ensured for all participants during the process. All data collection measures were kept in envelopes and the authors of the study had exclusive access to them.

### 2.5. Statistical Analysis

The statistical analysis was performed using the Statistical Package for the Social Sciences (SPSS, Inc., Chicago, IL, USA) for Windows, version 26.0. A bilateral contrast and a 95% level of confidence were adopted. A descriptive analysis was performed of all the variables gathered for each group. For this study, the means and standard deviations (SD) for each item were calculated as well as the bivariate correlations. Mean differences tests of the Satisfaction Scale Questionnaire items and domains were carried out to identify if the type of simulation affects satisfaction (Student’s *t* and analysis of variance (ANOVA)). The significance level was set at *p* < 0.05.

## 3. Results

In total, 393 nursing degree students participated in this study: 207 from the University of Cantabria (mean age 21.68, SD 4.46, 82.2% women) and 186 from the University of Seville (mean age 20.89, SD 3.36, 85.5% women). This questionnaire has presented a positive factorial structure, a good predictive capacity and a high internal consistency (alpha = 0.857).

Descriptive statistics of each item of the SSHF are presented in Table A1 (Appendix A) as well as the mean differences tests. In general terms, we see that the degree of satisfaction in almost all items is high (greater than 3), and slightly higher in MSF students, with statistically significant differences between both groups. This analysis is complemented by Table A2 (Appendix A), which shows the bivariate correlations of the items and the type of simulation. Then, statistically significant differences are reported for most items except item 3 “Cases recreated real situations”, item 7 “Clinical simulation is useful to assess a patient’s clinical situation”, item 11 “Simulation has made me think about my next clinical practice” and item 22 “I have improved communication with the family”. Students who carried out HFS did not always report the highest value in all the items.

The descriptive statistics and the ANOVA test are presented in Table 1. There was no significance difference between the two groups in age (*t* = 0.00, *p* < 0.05). The medium-fidelity group had higher scores in all the domains (except “Relationship between theory and practice”), and the differences are statistically significant. Furthermore, the highest value is achieved by both groups in the communication domain. In other words, they indicated that they are really satisfied with the improvement of their communication skills due to simulation practice.

In contrast, the lowest score in the MFS group was obtained in the domain “relationship between theory and practice”; that is, how case simulations worked when they are related to the theory learned. Regarding the HFS group, the lowest level of satisfaction was in the facilities and equipment domain.

## 4. Discussion

Beginner nursing students’ satisfaction about simulation experiences presented high scores in both cases (MFS and HFS). Different authors have also reported high scores, supporting our findings regarding the students’ satisfaction with both simulation methods [26,32,33,37]. The previous literature argues that this could be due to students forgetting disbelief before engaging in the task [38]. Another possible explanation for the satisfaction in this simulation learning is the importance of high intrinsic motivation for learning that students have [39].

In particular, our research goes further and analyses if there are differences in the level of satisfaction of beginner nursing students between the MFS group and the HFS group.

At this point, our results present a statistically significant difference, with the MFS group achieving a higher score than the HFS group. They also expressed significantly greater self-confidence dimension. Our results agree with Wang et al.’s [33] findings, which argued that this is explained by the fact that students are beginners with very little clinical experience, and this was their first exposure to both MFS and HFS.

It is possible that these results may be due to the fact that MFS ensures a greater satisfaction than HFS which is more complex for students, and therefore they prefer specific cases to real situations. The results seem to indicate that the group which received MFS was more satisfied with it. This could be due to their degree of satisfaction regarding their achievement being higher than in the cases presented with HFS when it comes to evaluating simpler competencies. The complexity of the cases and the technology itself are often a barrier to effective learning, as some studies indicate. In this sense, the literature suggests that simple scenarios, appropriate to the student’s degree of knowledge, which favour the acquisition of achievement as they advance in their training, seem more appropriate, provided they are the ones most adapted to the student’s level [40,41,42]. In this sense, other authors have reported that greater self-confidence and satisfaction with simulation has a protective impact on students’ anxiety levels [13,43], while increasing their confidence and self-efficacy during patient care. In this sense, it would be interesting to design a programme to reduce anxiety via simulation [44].

Benner [14] differentiated between theoretical and practical care and defends the fact that the latter may be developed even before theory, and it should be helpful to extend this [16]. In our case, although they have no clinical experience, the students have worked on their skills and abilities in an acute patient with LFS, and they report a higher satisfaction with MFS than with HFS, something similar to the evidence found by Basack in 2016 [45].

Some authors [32,33] also compared both levels of simulation practice; however, there were no statistically significant differences. Baptista et al. [26] reported that students’ satisfaction is higher with HFS, which may be due to the fact that they are students in their last year of training and they may be more satisfied with a more advanced level. On the other hand, Lapkin and Levett-Jones [32] analysed the level of satisfaction of beginner students considering the cost of medium- and high-fidelity mannequins. Their results show that significant differences do not exist between both methods, although the scores are higher for HFS. Consequently, they concluded that the medium-fidelity option is the most profitable approach because it only requires a fifth of the cost to obtain the same effect in satisfaction, clinical reasoning and acquisition of knowledge.

We believe the reason for these discrepancies may be due to the level of maturity of each student, whether he or she is a true novice or more experienced. Our results go along with Lubbers and Rossman’s evidence [46], since the findings suggest that beginner students’ satisfaction is higher with MFS.

### Limitations

The limitations of this study should be acknowledged. Firstly, the study was carried out with students who had little previous clinical experience and the results cannot be extrapolated to students with more clinical experience. In addition, the research has been performed in two different settings so the differences found could also be affected by the particularities of each setting. In this regard, future research should control the particularities of the setting to isolate the effect of the different types of simulation as much as possible. Finally, further research that goes beyond the differences in the level of satisfaction and considers the learning process in a more complete way is encouraged.

## 5. Conclusions

Nursing students involved in this research with no previous clinical experience who participated in an MFS showed a significantly higher level of satisfaction than those who enrolled in an HFS, although due to practice, both types of simulations enhanced student communication skills. The use of simulation, regardless of the degree of fidelity, is well-received by nursing students, who maintain high levels of satisfaction. It seems reasonable to recommend its use, in all degrees of fidelity (MFS, HFS), for the acquisition of competencies, since student satisfaction with their own performance is an element favouring their involvement in the learning process.

## Figures and Tables

**Table 1:** Mean differences test of Satisfaction Scale Questionnaire with High-Fidelity Clinical Simulation (SSHF) domains by type of simulation (medium- and high-fidelity simulation (MFS and HFS)).

SSHF Domains	Mean Value MFS (SD)	Mean Value HFS (SD)	Student-*t*
1. Simulation utility.	4.63 (0.47)	4.30 (0.45)	7.321 ***
2. Characteristics of cases and applications.	4.65 (0.51)	4.27 (0.54)	7.220 ***
3. Communication.	4.85 (0.52)	4.65 (0.49)	4.033 ***
4. Self-reflection on performance.	4.79 (0.43)	4.63 (0.41)	3.874 ***
5. Increased self-confidence.	4.50 (0.60)	4.25 (0.55)	4.487 ***
6. Relationship between theory and practice.	4.29 (0.64)	4.55 (0.42)	−4.850 ***
7. Facilities and equipment.	4.52 (0.61)	3.91 (0.60)	5.709 ***
8. Negative aspects of simulation.	4.61 (0.779)	4.19 (0.86)	7.306 ***

Levene´s test was carried out for each domain and the appropriate mean difference test was carried out. SD = Standard deviation. Significance level: *** *p*-value < 0.005.

## Data Availability

Data is contained within the article or supplementary material.

## Appendix A

**Table A1:** Mean differences test of SSHF items by type of simulation (MFS and HFS).

SSHF Items	Mean Value MFS (SD)	Mean Value HFS (SD)	Student’s *t*
1. Facilities and equipment were real	4.15 (0.927)	4.48 (0.606)	−4.293 ***
2. Objectives were clear cases	4.70 (0.693)	4.31 (0.651)	6.154 ***
3. Cases recreated real situations	4.65 (0.717)	4.73 (0.488)	−1.237
4. Timing for each simulation case was adequate	3.38 (1.349)	4.24 (0.790)	−7.822 ***
5. The degree of case difficulty was appropriate for my knowledge	4.41 (0.790)	4.03 (0.799)	4.988 ***
6. I felt comfortable and respected during the sessions	4.82 (0.665)	4.41 (0.800)	5.831 ***
7. Clinical simulation is useful to assess a patient’s clinical situation	4.67 (0.720)	4.63 (0.585)	0.648
8. Simulation practices help you learn to avoid mistakes	4.68 (0.684)	4.36 (0.764)	4.496 ***
9. Simulation has helped me to set priorities for action	4.83 (0.487)	4.59 (0.487)	4.817 ***
10. Simulation has improved my ability to provide my patients with care	4.75 (0.584)	4.40 (0.623)	5.988 ***
11. Simulation has made me think about my upcoming clinical practice	4.70 (0.573)	4.61 (0.576)	1.706
12. Simulation improves communication and teamwork	4.76 (0.570)	4.49 (0.685)	4.479 ***
13. Simulation has made me more aware/concerned about clinical practice	4.61 (0.779)	4.19 (0.868)	5.275 ***
14. Simulation is beneficial to relate theory to practice	4.87 (0.592)	4.70 (0.513)	3.104 ***
15. Simulation allows us to plan patient care effectively	4.59 (0.662)	4.11 (0.719)	7.102 ***
16. I have improved my technical skills	4.71 (0.685)	4.07 (0.835)	8.888 ***
17. I have reinforced my critical thinking and decision-making	4.74 (0.597)	4.25 (0.676)	8.148 ***
18. Simulation helped me assess a patient’s condition	4.67 (0.615)	4.33 (0.632)	8.148 ***
19. This experience has helped me prioritise care	4.68 (0.643)	4.36 (0.652)	5.619 ***
20. Simulation promotes self-confidence	4.46 (0.830)	4.17 (0.856)	3.439 ***
21. I have improved communication with the team	4.66 (0.681)	4.20 (0.775)	6.700 ***
22. I have improved communication with the family	3.97 (1.304)	4.01 (0.798)	−0.410
23. I have improved communication with the patient	4.40 (0.823)	4.23 (0.707)	2.268 *
24. This type of practice has increased my security	4.46 (0.783)	4.01 (0.919)	5.422 ***
25. I was thrown off balance during some of the cases	4.17 (1.044)	3.51 (1.189)	6.226 ***
26. Interaction with simulation has improved my clinical competence	4.64 (0.647)	4.23 (0.660)	6.409 ***
27. The teacher gave constructive feedback after each session	4.93 (0.542)	4.69 (0.540)	4.579 ***
28. Debriefing has helped me reflect on the cases	4.81 (0.629)	4.68 (0.585)	2.242 *
29. Debriefing at the end of the session has helped me correct mistakes	4.84 (0.616)	4.60 (0.629)	4.000 ***
30. I found out about the cases’ theoretical side	4.45 (0.752)	3.74 (0.831)	9.192 ***
31. I have learned from the mistakes I made during the simulation	4.83 (0.570)	4.51 (0.612)	5.770 ***
32. Practicality	4.84 (0.604)	4.69 (0.540)	2.636 **
33. Overall satisfaction with the sessions	4.72 (0.640)	4.58 (0.588)	2.403 *

Levene´s test was carried out for each item and the appropriate mean difference test was carried out. SD = Standard deviation. Significance level: *** *p*-value < 0.005, ** *p*-value < 0.01, * *p*-value < 0.05.

**Table A2:** Bivariate correlations of type of simulation and SSHF items.

SSHF Items	Pearson Correlation Coefficient	SSHF Items	Pearson Correlation Coefficient
1. Facilities and equipment were real	0.213 ***	18. Simulation helped me assess a patient’s condition	−0.259 ***
2. Objectives were clear cases	−0.281 ***	19. This experience has helped me prioritise care	−0.239 ***
3. Cases recreated real situations	0.063	20. Simulation promotes self-confidence	−0.164 ***
4. Timing for each simulation case has been adequate	0.375 ***	21. I have improved communication with the team	−0.297 ***
5. The degree of case difficulty was appropriate to my knowledge	−0.232 ***	22. I have improved communication with the family	0.021
6. I felt comfortable and respected during the sessions	−0.260 ***	23. I have improved communication with the patient	−0.110 *
7. Clinical simulation is useful to assess a patient’s clinical situation	−0.031	24. This type of practice has increased my security	−0.250 ***
8. Simulation practices help you learn to avoid mistakes	−0.207 ***	25. I was thrown off balance during some of the cases	−0.279 ***
9. Simulation has helped me to set priorities for action	−0.218 ***	26. Interaction with simulation has improved my clinical competence	−0.292 ***
10. Simulation has improved my ability to provide my patients with care	−0.271 ***	27. The teacher gave constructive feedback after each session	−0.213 ***
11. Simulation has made me think about my next clinical practice	−0.081	28. Debriefing has helped me reflect on the cases	−0.108 *
12. Simulation improves communication and teamwork	−0.203 ***	29. Debriefing at the end of the session has helped me correct mistakes	−0.186 ***
13. Simulation has made me more aware/concerned about clinical practice	−0.239 ***	30. I found out about the cases’ theoretical side	−0.401 ***
14. Simulation is beneficial to relate theory to practice	−0.149 ***	31. I have learned from the mistakes I made during the simulation	−0.262 ***
15. Simulation allows us to plan patient care effectively	−0.319 ***	32. Practicality	0.127 **
16. I have improved my technical skills	−0.379 ***	33. Overall satisfaction of sessions	−0.114 *
17. I have reinforced my critical thinking and decision-making	−0.355 ***		

Significance level: *** *p*-value < 0.005, ** *p*-value < 0.01, * *p*-value < 0.05.

## References

[1] National Agency for the Evaluation of Quality and Accreditation Support Guide for the Writing, Implementation and Evaluation of Learning Outcomes Version 1.0. http://www.aneca.es/Sala-de-prensa/Noticias/2013/ANECA-presenta-la-Guia-para-la-redaccion-y-evaluacion-de-los-resultados-del-aprendizaje.

[2] ORDEN CIN/2134/2008, 3 July. Ministry of Science and Innovation. Spain. https://www.boe.es/diario_boe/txt.php?id=BOE-A-2008-12388.

[3] Kajander-Unkuri S., Meretoja R., Katajisto J., Saarikoski M., Salminen L., Suhonen R., Leino-Kilpi H. (2014). Self-assessed level of competence of graduating nursing students and factors related to it. Nurse Educ. Today.

[4] Stevanin S., Bressan V., Bulfone G., Zanini A., Dante A., Palese A. (2015). Knowledge and competence with patient safety as perceived by nursing students: The findings of a cross-sectional study. Nurse Educ. Today.

[5] Deschênes M.F., Goudreau J., Fernandez N. (2020). Learning strategies used by undergraduate nursing students in the context of a digitial educational strategy based on script concordance: A descriptive study. Nurse Educ. Today.

[6] Gilliam P., Pabst P., Spencer S. (2013). Making high-fidelity simulation relevant to the home setting. Nurse Educ..

[7] Badir A., Zeybekoglu Z., Karacay P., Göktepe N., Topcu S., Yalcin B., Kebapci A., Oban G. (2015). Using high-fidelity simulation as a learning strategy in an undergraduate intensive care course. Nurse Educ..

[8] Raurell-Torredà M., Olivet-Pujol J., Romero-Collado À., Malagon-Aguilera M.C., Patiño-Masó J., Baltasar-Bagué A. (2015). Case-based learning and simulation: Useful tools to enhance nurses’ education? Nonrandomized controlled trial. J. Nurs. Scholarsh..

[9] Bruce R., Levett-Jones T., Courtney-Pratt H. (2019). Transfer of learning from university-based simulation experiences to nursing students’ future clinical practice: An exploratory study. Clin. Simul. Nurs..

[10] Ricketts B. (2011). The role of simulation for learning within pre-registration nursing education—A literature review. Nurse Educ. Today.

[11] Thidemann I.J., Söderhamn O. (2013). High-fidelity simulation among bachelor students in simulation groups and use of different roles. Nurse Educ. Today.

[12] Tuzer H., Dinc L., Elcin M. (2016). The effects of using high-fidelity simulators and standardized patients on the thorax, lung, and cardiac examination skills of undergraduate nursing students. Nurse Educ. Today.

[13] Doğru B.V., Aydın L.Z. (2020). The effects of training with simulation on knowledge, skill and anxiety levels of the nursing students in terms of cardiac auscultation: A randomized controlled study. Nurse Educ. Today.

[14] Benner P. (1984). From Novice to Expert: Excellence and Power in Clinical Nursing Practice.

[15] Benner P., Sutphen M., Leonard V., Day L. (2010). Educating Nurses: A Call for Radical Transformation.

[16] Alligood M.R. (2018). Nursing Theorists and Their Work.

[17] Jeffries P.R. (2005). A framework for designing, implementing, and evaluating simulations used as teaching strategies in nursing. Nurs. Educ. Perspect..

[18] Alinier G. (2007). A typology of educationally focused medical simulation tools. Med. Teach..

[19] Guinea S., Andersen P., Reid-Searl K., Levett-Jones T., Dwyer T., Heaton L., Flenady T., Applegarth J., Bickell P. (2019). Simulation-based learning for patient safety: The development of the Tag Team Patient Safety Simulation methodology for nursing education. Collegian.

[20] (2011). The International Nursing Association for Clinical Simulation and Learning Board of Directors. Standard I: Terminology. Clin. Simul. Nurs..

[21] Decker S., Sportsman S., Puetz L., Billings L. (2008). The evolution of simulation and its contribution to competency. J. Contin. Educ. Nurs..

[22] Kirschner P.A., van Merriënboer J.J. (2013). Do learners really know best? Urban legends in education. Educ. Psychol..

[23] Oosterheert I., Meijer P.C., van der Neut I. (2020). Towards Broader Views on Learning to Teach: The Case of a Pedagogy for Learning to Teach for Creativity. Education beyond Crisis.

[24] Zapko K.A., Ferranto M.L.G., Blasiman R., Shelestak D. (2018). Evaluating best educational practices, student satisfaction, and self-confidence in simulation: A descriptive study. Nurse Educ. Today.

[25] Robinson B.K., Dearmon V. (2013). Evidence-based nursing education: Effective use of instructional design and simulated learning environments to enhance knowledge transfer in undergraduate nursing students. J. Prof. Nurs..

[26] Baptista R.C., Paiva L.A., Gonçalve R.F., Oliveira L.M., Pereira M.F., Martins J.C. (2016). Satisfaction and gains perceived by nursing students with medium and high-fidelity simulation: A randomized controlled trial. Nurse Educ. Today.

[27] Kim S.S., Kim E.J., Lim J.Y., Kim G.M., Baek H.C. (2018). Korean Nursing Students’ Acquisition of Evidence-Based Practice and Critical Thinking Skills. J. Nurs. Educ..

[28] Cason M., Atz T., Horton L.F. (2017). New nursing graduates’ self-efficacy ratings and urinary catheterization skills in a high-fidelity simulation scenario. Clin. Simul. Nurs..

[29] Al-Ghareeb A.Z., Cooper S.J. (2016). Barriers and enablers to the use of high-fidelity patient simulation manikins in nurse education: An integrative review. Nurse Educ. Today.

[30] Cant R.P., Cooper S.J. (2010). Simulation-based learning in nurse education: Systematic review: Simulation in nursing. J. Adv. Nurs..

[31] Tosterud R., Hedelin B., Hall-Lord M.L. (2013). Nursing students’ perceptions of high- and low-fidelity simulation used as learning methods. Nurse Educ. Pract..

[32] Lapkin S., Levett-Jones T. (2011). A cost-utility analysis of medium vs. high-fidelity human patient simulation manikins in nursing education. J. Clin. Nurs..

[33] Wang A.L., Fitzpatrick J., Petrini M.A. (2013). Comparison of two simulation methods on Chinese BSN students’ learning. Clin. Simul. Nurs..

[34] Schulz K.F., Altman D.G., Moher D. (2010). CONSORT 2010 statement: Updated guidelines for reporting parallel group randomised trials. BMJ.

[35] Levett-Jones T., Lapkin S. (2014). A systematic review of the effectiveness of simulation debriefing in health professional education. Nurse Educ. Today.

[36] Alconero-Camarero A.R., Gualdrón-Romero A., Sarabia-Cobo C.M., Martínez-Arce A. (2016). Clinical simulation as a learning tool in undergraduate nursing: Validation of a questionnaire. Nurse Educ. Today.

[37] Johnston S., Parker C.N., Fox A. (2017). Impact of audio-visual storytelling in simulation learning experiences of undergraduate nursing students. Nurse Educ. Today.

[38] Herrington J., Oliver R., Reeves T.C. (2003). Patterns of engagement in authentic online learning environments. Australas. J. Educ. Technol..

[39] Martens R., Gulikers J., Bastiaens T. (2004). The impact of intrinsic motivation on e-learning in authentic computer tasks. J. Comput. Assist. Learn.

[40] Larew C., Lessans S., Spunt D., Foster D., Covington B.G. (2006). Innovations in clinical simulation: Application of Benner’s theory in an interactive patient care simulation. Clin. Simul. Nurs..

[41] Alfes C.M. (2011). Evaluating the use of simulation with beginning nursing students. Nurse Educ..

[42] Kang K.-A., Kim S.-J., Lee M.-N., Kim M., Kim S. (2020). Comparison of Learning Effects of Virtual Reality Simulation on Nursing Students Caring for Children with Asthma. Int. J. Environ. Res. Public Health.

[43] Szpak J.L., Kameg K.M. (2013). Simulation decreases nursing student anxiety prior to communication with mentally ill patients. Clin. Simul. Nurs..

[44] Khalaila R. (2014). Simulation in nursing education: An evaluation of student’s outcomes at their first clinical practice combined with simulation. Nurse Educ. Today.

[45] Basak T., Unver V., Moss J., Watts P., Gaioso V. (2016). Beginning and advanced students’ perceptions of the use of low- and high-fidelity mannequins in nursing simulation. Nurse Educ. Today.

[46] Lubbers J., Rossman C. (2017). Satisfaction and self-confidence with nursing clinical simulation: Novice learners, medium-fidelity, and community settings. Nurse Educ. Today.

